# After neonatal care, what next? A qualitative study of mothers’ post-discharge experiences after premature birth in Kenya

**DOI:** 10.1186/s12939-024-02340-y

**Published:** 2025-01-20

**Authors:** Justinah Maluni, Dorothy Oluoch, Sassy Molyneux, Mwanamvua Boga, Caroline Jones, Florence Murila, Mike English, Sue Ziebland, Lisa Hinton

**Affiliations:** 1 Health Services Unit, KEMRI-Wellcome Trust Research Programme, P.O Box, Nairobi, 43640-00100 Kenya; 2 Nuffield Department of Medicine, University of Oxford, University of Oxford, Old Road Campus, Oxford, OX3 7BN UK; 3 Department of Paediatrics and Child Health, University of Nairobi, P. O. Box 19676, Nairobi, Kenya; 4 Nuffield Department of Primary Care Health Sciences, University of Oxford, Radcliffe Observatory Quarter, Woodstock Road, Oxford, OX2 6GG UK

**Keywords:** Post-discharge, Mothers’ experiences, Premature birth, Newborn, Low-income setting, Information and support, Stigma

## Abstract

**Background:**

Approximately 15 million babies are born prematurely every year worldwide. Sub-Saharan Africa (SSA) and Asia account for more than half of the global preterm deliveries. Prominent healthcare structural and socio-economic factors in SSA, for example poverty and weak health systems, amplify vulnerabilities for mothers and premature babies; often leading to poor outcomes. Post-discharge mortality rates are high, and readmission is common. For mothers of premature babies, the transition home from hospital is marked by challenges and uncertainties. This study explored the post-discharge experiences of mothers of premature babies with the aim of identifying their needs and suggests strategies to strengthen and support their discharge preparation to care for their premature baby at home, and to and reduce mortality and readmission rates.

**Methods:**

Narrative interviews were conducted face-to-face in English or Swahili with 34 mothers of premature babies recruited from two public hospitals and a social support group in Nairobi, Kenya between August—November 2021. Interviews were audio and video-recorded and transcribed for analysis. After transcription, the interviews were translated, where applicable, and thematic analysis was undertaken.

**Results:**

For mothers of premature babies, discharge from neonatal care and the transition home is a complex process marked with mixed emotions; many reported feeling unprepared and facing stigma while in hospital and in their communities. Mothers described the emotional challenges of discharge from the neonatal unit and their information and support needs. Minimal involvement in their baby’s care while in the neonatal unit appeared to contribute to the mothers’ lack of confidence in caring for their babies independently post-discharge when they no longer had the support of the clinical and nursing staff. Insufficient information provided on discharge hindered a smooth transition to home, highlighting the need for information to support mothers’ confidence after discharge. Stigma relating to beliefs around preterm births was experienced by some of the mothers in the community and within some health clinics.

**Conclusions:**

To support transitions home, strengthening the timing and adequacy of information provided to mothers at discharge from the neonatal unit in low-income settings in SSA and Asia – such as Kenya—is essential. Introducing strategies to build and assess mothers’ competencies with skills such as breastfeeding and identifying signs of deterioration before discharge could support their smooth transition home. Targeted engagement interventions at the community level could demystify and address stigma and knowledge gaps about premature deliveries at the community and social levels more broadly and within the health system.

**Supplementary Information:**

The online version contains supplementary material available at 10.1186/s12939-024-02340-y.

## Background

Approximately 15 million babies are born prematurely every year, and prematurity rates are increasing in almost all countries with reliable data [1]. Births to mothers in sub-Saharan Africa and Asia account for over 60% of premature deliveries worldwide [2]. Prematurity is the leading cause of death in children under the age of 5 years; one million children die each year due to complications of preterm birth [3]. Many surviving preterm babies are at risk of malnutrition and anaemia and therefore may face a lifetime of disability, including learning disabilities, visual and hearing problems [4]. Evidence from a large cohort of babies with diverse racial and sociodemographic backgrounds in the USA showed that out of 1000 extremely low birth weight (ELBW) babies that died, approximately 2.23% of them died after discharge from the neonatal intensive care unit (NICU) [5]. Many low-level hospitals in low-/middle income countries or low clinical resource settings do not have NICUs and have a more basic focus such as sip feeding, kangaroo care and oral iron replacement. ELBW babies and very preterm (born between 28 and 32 weeks) require repeated close monitoring, and regular follow-up visits are recommended. However, as highlighted by Wainaina et.al, in a retrospective quantitative study that aimed to quantify post-discharge needs in Kenya, specialist services are not available across many facilities, and these babies are often referred to a general paediatric or neonatal outpatient clinic which lacks multi-professional team support. Post-discharge follow-up is also not adequately coordinated through community-run clinics.[6].

ELBW babies and preterms born between 28 and 32 weeks may have multiple complications and require inpatient neonatal care for many weeks. Studies from low and high-income settings about parents’ and mothers’ experiences of in-hospital care for small and sick newborns reveal challenges such as a lack of information [7], low levels of involvement in the care of their baby [8], and mothers’ negative interactions with the neonatal staff [9]. These reported issues can lead to emotional distress for mothers during their baby’s hospitalisation [10]. Furthermore, the delivery of a preterm baby offers a distinctive challenge to a mother, for which she is most often unprepared [11]. Maternal competence and confidence play a significant role in the adjustment to motherhood; a new mother must be able to provide care and foster growth and development in her infant [12]. The development of maternal competence can be delayed by lengthy hospitalisation, resulting in mothers feeling ill-prepared to take care of their preterm baby at home [13]. The prolonged hospital stay and complexities surrounding the care of preterm babies can increase anxiety and fears [11, 14]. One of the strategies commonly adopted in high-income countries (HIC) to prepare caregivers for discharge home is ‘rooming-in’, where a mother is allowed to stay with their baby, with nurses observing and supporting the mother’s skills in taking care of their baby [15–17]. Previous ethnographic research in neonatal units in public hospital settings in Kenya has reported the norm of sharing or task-shifting to mothers [8]. Assessing a mother’s readiness, capabilities and confidence to take on caring tasks is rare in low-income settings.

Studies conducted in HICs, defined as those with a Gross National Income (GNI) per capita, calculated using the World Bank Atlas methods, of more than $ 14,005 in 2023 [18] have reported post-discharge challenges that place babies at risk. These include their nutrition and irregular sleep patterns as well as the parents’ physical and psychological exhaustion [19] and stress [19–21]. Quantitative evidence from HICs report that mothers experience fear, depression [22] and isolation [23]. Once home, some parents become overwhelmed by their baby’s health problems and often only see doctors unfamiliar with their baby’s history and condition [24]. Research undertaken in the USA has shown that mothers who experience extreme levels of anxiety, distress, or depression while their baby is in the neonatal unit have a negative perception towards their child’s abilities and strengths at one year of age and experience psychological distress a year post-discharge [25]. An Australian study reported approximately 20% of parents with children born very prematurely have post-traumatic stress symptoms two years post-partum [26]. In high and low-resource settings, these factors are compounded by financial stresses originating from high medical bills for childbirth, neonatal care and re-hospitalisation [24, 27] and non-medical issues such as job loss, reduced income and employment options [24, 28].

Postnatal care (PNC) is critical for mothers and their newborns[29–31] and research in sub-Saharan Africa suggests information and preparation for discharge improves self-assurance and capacity to offer care [32]. Therefore, mortality, morbidity, and the risk of hospital readmissions are likely to decrease by improving post-discharge care [29, 33]. However, there is limited published research from low-income settings on the post-discharge needs of families of preterm babies including gaps in evidence and policy on contextually appropriate interventions to help families cope with the post-discharge challenges that contribute to mortalities [34]. This paper describes a qualitative study of experiences of mothers of premature babies after discharge from the NICU in two public and four private hospitals in Kenya. The paper endeavours to understand their support and information needs and how to best prepare them for their baby’s discharge.

## Methods

### Aim and design

The findings reported here form part of a larger project, *Exploring the potential for using parent experiences of pre-term birth to improve care in LMICs,* which ran from 2020–2023. This study examined the feasibility of capturing mothers’ experiences of hospitalised pre-term babies through narrative research interviews (video-recorded with consent) and explored the potential of the potential for using parent experiences to enhance existing educational tools to improve patient-centred care in Kenya to improve patient-centred care in Kenya. The results of the evaluation will be published elsewhere. In the narrative interviews which we report here, we explored the experiences of mothers of hospitalised pre-term babies during admission and post-discharge. In this paper, we report on the mothers’ experiences after discharge from the hospital.

### Study setting

The study was conducted in Nairobi County, Kenya, between September 2021 and November 2022. Participants were recruited from two public hospitals in the County and via a Kenyan-based support group for mothers who had given birth prematurely. The first study hospital was a county referral hospital with a bed capacity of about 60 and offering care to mothers and sick and small newborns. The second hospital has a higher bed capacity of about 150 offering specialised neonatal unit care. The support group established by mothers who themselves had the experience of preterm delivery offers support to mothers of preterm babies, with membership spread across the country. Mothers from the support group were cared for in private and public facilities (see Supplementary material 1).

### Data collection

Data were collected through narrative interviews with purposively selected mothers who had a premature baby within the previous two years and whose baby was alive at the time of the interview. The interviews were conducted face-to-face by DO or JM, both fluent in English and Swahili. Due to the COVID-19 pandemic, health and safety measures did not permit the study teams to visit people’s homes, and as such, interviews were scheduled in a hotel setting following COVID-19 safety protocols. The interviews were conducted in English or Swahili depending on the participant’s preference. Interviews used a narrative approach, starting with an open question inviting mothers to share their experiences [35]. These open narratives were followed up with a handful of semi-structured questions using a topic guide developed by DO and LH and refined by co-authors. During the interviews mothers were invited to relate their experiences of their baby’s admission and care on the newborn unit as well as their experiences after discharge.

### Data analysis

Interviews were transcribed verbatim and translated into English, where applicable, by third-party transcribers. Transcripts were verified by JM. A thematic analysis was undertaken using the one sheet of paper (OSOP) method [35], based on a modified grounded theory and constant comparison. A coding framework was developed by DO, LH and JM based on the themes derived from the interview guide and those emerging from the data. The data were managed and analysed in NVIVO 12 Pro.

### Ethical approval

The study was conducted in compliance with the Helsinki Declaration. Ethical approval was granted by the Scientific Ethical Review Committee of the Kenya Medical Research Institute (Protocol no. KEMRI/SERU/CGMR-C/244/4180) and the Cambridge Psychology Research Ethics Committee (PRE. 2021.052). In addition, permission to conduct the study was obtained from the National Commission for Science and Technology (NACOSTI) and from the Nairobi County Government as well as each hospital’s research department, the hospital medical superintendent and the neonatal unit in-charges. Written informed consent was obtained from the study participants after explaining the purpose of the study and giving them the opportunity to ask questions. The study team included experienced social scientists in the UK and Kenya (English and Swahili speakers), a paediatrician and a nurse based in Kenya.

## Results

Thirty-four mothers who had given birth to a preterm baby in hospitals within Nairobi County and whose babies’ age was between 3 months and 2 years participated in the narrative interviews. Their socio-demographic data are presented in Supplementary Material 1.

**Fig. 1:**
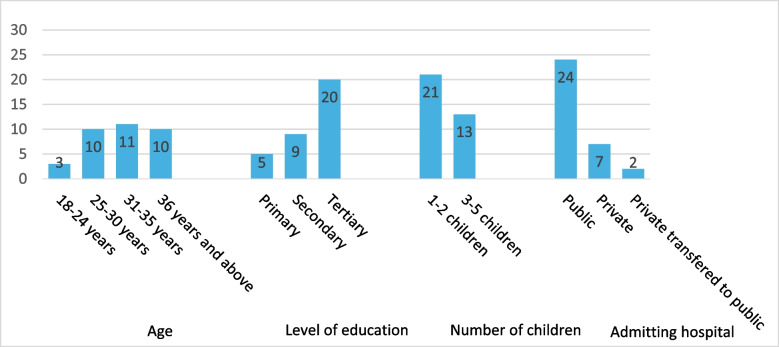
Demographic characteristics of mothers to preterm babies in hospitals in Nairobi, Kenya (*n* = 34)

Of those interviewed, eleven mothers had given birth in hospital one (HF1), a county referral public hospital, eleven in hospital two (HF2), a larger public hospital, and twelve were from a support group (SG) and had given birth in either a subcounty or county referral public hospital or private hospitals in Nairobi (Fig. 1).

Twenty interviews were conducted in Swahili and 14 in English. Here we report on the key themes identified from the narratives. These relate to feelings during discharge; information provision and information needs; experiences and challenges once home; and stigma.

### Feelings during discharge: excitement and fear

In their narratives, mothers reported that for a preterm baby to be discharged, their weight needed to reach 1.8 kg. The babies’ weights were measured and recorded daily. In addition to their weight, the baby’s health condition had to have improved for them to be discharged. Mothers judged this improvement in their baby’s condition in relation to being weaned off machines and transferred from critical care rooms to the spaces/rooms to which stable babies were admitted. Having experienced the challenges of being a mother on the neonatal unit, discharge home was eagerly anticipated.

*“I was happy. Just when the baby reached, because you are told when the baby reaches 18, 1.8, you just leave. So, when my baby reached 1.8, I started calling, even before the doctor had told me you were supposed to go home. I started making calls going, "Come for me tomorrow the baby has attained the required kilo for leaving”. ****HF1_007***

Mothers had stayed in the hospital with their premature baby for periods ranging from one week to over 3 months. One mother described it as being ‘in prison’; thus the possibility of discharge was often met with joy and relief. Mothers’ descriptions of their hospital stay revealed multi-layered difficulties including regulated access to the newborn unit, strict timings for tasks such as feeding and top tailing (washing their face, neck, hands and bottom) and at times lack of comfort related to a lack of sufficient facilities, for example, adequate beds or crowding on the wards.

“*“I was happy, I was happy finally, you know I used to tell myself, “I can’t wait for the day…” You see ****#name of private hospital mentioned**** you are usually escorted by the nurse carrying the baby then the car is usually at the entrance…that used to be my dream. I used to figure myself leaving with my baby” ****SG_02***

However, these feelings of joy could be accompanied by fear and anxiety. Mothers often expressed concern about how tiny and vulnerable their baby still was.

*“You don’t know what you are feeling at that point. It was the… okay, the thing is, it comes with fear, you are excited, you really want to go home, but this child is still tiny, because their discharge weight is 1.75 (Kg) up to 2kgs depending on if the baby is feeling good. So, if the baby is stable… mine was born at 1700 grams. What are you going to do with this baby? You know, here you were confident, if he vomits a little, “doctor the baby is vomiting”, he tells you, “aahh… mum bend the baby and do this”. Now you’ve been told to go home, you will be alone, what will happen? ****HF2_001***

This anxiety and fear of the reality of the transition home was expressed in many forms. Mothers whose babies had experienced complications while in the neonatal unit worried about the re-emergence of those complications post-discharge. These fears were exacerbated by the reality of being on their own without clinical support or near the healthcare staff who supported them in hospital. Some doubted their ability to care for their baby at home even if they were not a first-time parent.

*“At first, I was anxious. You know this is life outside and you will not have any doctor to consult. I was very anxious, especially on the first day at home, the baby cries. You don't know what you will do”. ****HF1_05***

Mothers reported fear and anxiety about the baby’s condition post-discharge. These fears were heightened after weeks of watching their baby struggle for life in neonatal care.

*“How will we handle life after…?” Reason being you have spoken to other mothers, and they have told you the challenges they have gone through: the challenges of not feeding, unable to feed the baby, challenges of the baby getting apneic attacks, we were also told challenges of those babies getting rickets because we tend to make them stay inside. You don’t want to expose them. So, I had fears. I had so much fear.” ****SG_02***

In addition, many preterm babies had been dependent on medical interventions while in the neonatal unit, for example, nasogastric tube feeding, incubators and controlled temperature environments, and would now need to survive without these. Mothers were reassured by seeing their babies progressing well and being weaned off machines. But they also described their uncertainties about the realities of being able to provide adequate care and the transition from the hospital setting to their home.

*“…At the hospital he is used to that, if not an incubator, there is a heater. You’ve gone and they are telling you just continue with kangaroo. So, there are those fears. There is that excitement that you really want to go, you know the hospital is like a mini prison, but now you are going home with this baby! It was mixed feelings because you have fear.” ****HF2_001***

### Information provided and needed at discharge

Mothers reported receiving information on various issues at discharge, including keeping their baby warm, maintaining hygiene and feeding, the importance of follow-up visits, kangaroo mother care (skin-to-skin contact with the baby) and the danger signs to look out for (including, for example jaundice, vomiting, fevers, failure to breastfeed). Table 1 provides a summary of the range of information the mothers reported receiving at discharge.

**Table 1:** Information provided to mothers at discharge

*Information provided*	*Illustrative quotations*
*Keeping the baby warm*	*“They just told me if your child has reached 1.8, you go home. And you buy that medication. If you don't have, you can't go home. And then I bought it. And then we went, they explained to us. If you see the child is too hot, you bring them to the hospital. If they are too cold, you bring them to the hospital. If they are crying a lot, you bring them to the hospital. Yes, that was what they told us”. ****HF1_002***
*Hygiene and feeding*	*“He told me first it was cleanliness. Second, the doctor told me not to leave the baby to suckle on their own because they don't have enough strength to suckle and get filled. Sometimes they will be suckling and sleep and think they are full but they are just tired. If they suckle too much, they get exhausted. So, they told me to go look for a cup for a baby. When the time reaches, I express and give it to them. The next round I breastfeed them, if I stay for a little while I express for them and make sure they are clean and where they are staying is clean, including the cup you are milking for them”. ****HF1_003*** *“Most of the counselling was being done in the morning, that morning, the way to breastfeed your child, you see now you’ve left, you are not feeding the child using a tube, and remember, he is small, and he hasn’t known how to suck the milk by himself, but now he has attained the kilos of sucking milk, so you feed as often as possible. If it is, you feed many times, many times ****[baby mumbling]**** many times, so that he may also grow. That feeding also helps, and then you are also told, issues concerning drinking water. After being released, that is the discharge now, you don’t need to be told much, they will repeat. But you already know, because you’ve stayed there for over ten days, and the topic is one, or two, mother’s health, child health, every day, you can only be a joker if you don’t”. ****HF1_004***
*Follow up visits and kangaroo mother care*	*“… but you are told, “kindly remember to attend the clinics”, two weeks has found us here, so you will not go the two weeks one, you will stay and come back… you will go the other clinic and it is okay and make sure you attend clinics”. ****HF1_004*** *“We were told to keep the baby well and keep her warm. To Kangaroo the babies and breastfeed them well… They were also to be exposed under sun, things like those. ****[baby crying]**** and to give the supplements that they had prescribed”. ****HF1_009*** *“Yeah, they explained to us on how to handle to the baby well at home. Especially insisting that we go on with kangaroo mother care. They told us, it’s not like we’ve been discharged from the hospital to go home and stay a normal life. You have to give your baby time, until they get to those kgs and kangaroo mother care, helps the baby in gaining weight fast. So, it was your hard work, the more you do it the better and the easier it will be for you”. ****HF2_001***
*Danger signs*	*“They said, in case the baby is not suckling, they will turn pale or yellowish, you are supposed to return to the hospital, to take him under sunlight, and follow up on immunization”. ****HF1_005*** *“Like if the baby has refused to breastfeed, because they are not eating any other food, they must breastfeed. If I see like they have started to change, instead of being pink, they start becoming white, they do not have blood, I should take them back, if they have started being yellow, if the eyes, if I look at the eyes and see that it is like I don’t know how they look like, like these palms, they are whitish or yellow. The other one was if the baby refused to breastfeed, or they are crying or they have fever”. ****HF2_008*** *“Yeah, we were told if there is anything abnormal, like if a baby convulses, if the fever is too high or the temperature drop very low. For pre-terms, their temperatures drop mostly, if the temperatures drop very low, we should go back to the hospital, or if they go so high and the baby convulses, we should go back to the hospital”. ****HF2_002*** *“Yeah, we were told if we see that the baby has fever, fever, we should take the baby back to the hospital, or diarrhea, and vomiting, we should take the baby back to the hospital”. ****HF2_004***

More than half the mothers felt that the information provided was insufficient. They, mentioned the need for more clarity on a health problem their baby had at discharge or may develop post-discharge, for example, reflux, and the ongoing needs of the baby. This added to their anxiety.

*“The fact that they didn't tell us the baby had that condition of refluxing, then we go, we don't know how to handle a refluxing baby, you don't even know whether the baby is refluxing in the first place. […..] Now you are constantly worried, “Is this child, okay? Is, is this child going to be, okay? Like, did I get a child from hospital to come and have issues at home” ****SG_10***

*“Yes, even at the point of discharge. No one told you. So, when you get home, give this baby porridge, this kind of food, no. You just sit there. Google, ask other moms now in the groups. When did you start giving your baby these? Now some mom say this, As some mom say, you know, now every mom has their own experience. Yeah. So, you don't even know will the baby choke.” ****SG_12***

### Experiences, and challenges once home

#### Identifying danger signs and re-hospitalisation

Once they had brought their babies home, the mothers described how they worried about the possibility of their baby suffering health problems or complications, especially at times when access to a health facility could be challenging. Some mothers described a lack of confidence in being able to identify danger signs and their ability to know whether to take the baby to the hospital.

*“On day two I saw something funny; the baby was bluish. I told the father who told me not to panic. In the night the baby was not okay. On the following day, the baby was just flat. The baby goes off and then the father tells me, “Why are you allowing the child to act as if dead? What is happening with you?” Then he told me that he is taking the baby to the sun but inside me I was not settled. I told him that the baby is not okay, the baby would turn blue and then regain consciousness. It is like she is dying and resurrecting.”* **SG_04**

Some of the mothers reported their babies being re-hospitalised post-discharge for jaundice, sepsis or laser treatment to prevent blindness. Re-hospitalisation of these premature babies after discharge becomes even more complex for mothers of twins.

*“... we were admitted twice then again for laser because [the] doctor said that is the only way by which we can prevent the blindness because that is what they would tend to get if you don’t treat it, so we had to do laser. Then after that, we were admitted again with sepsis tonsillitis; that is, I think back in May this year. Then actually she was discharged yesterday so I was about to cancel this interview.” ****SG_02***

*“After two weeks one baby developed jaundice. It was high but not that much, we had to go back to the hospital. The other baby developed jaundice after two days also. The scar was not healed and now I go back to the hospital and get admitted for two weeks. It was hard but I thank God they healed well. It was hard because the time I got admitted one baby had jaundice and the other one did not have. So, one was undergoing phototherapy, the other one is crying, the other one undergoing phototherapy is also crying. The other mothers felt pity for me, but the baby got well. After two days the other baby got sick. We had to stay in the hospital for almost two weeks.” ****HF2_011***

#### Feeding

Looking after a premature baby at home post-discharge came with many hurdles. One key example was finding the time and space to express milk with minimal support. While in hospital, the mothers appreciated the camaraderie between mothers going through the same situation. Moving back home, they were often isolated from other mothers and health professionals. This complicated the transition and their confidence in looking after the baby and performing key tasks.

*“The challenge was about expressing, because you know in the hospital all of you are expressing, but here you are alone, and you must express”. ****HF1_008***

In addition to expressing milk, there were often challenges with breastfeeding. Some of the mothers reported their babies not being able to attach and suckle as required, an indication that this had not been established nor assessed at the point of discharge. For some this prompted them to seek breastfeeding support back at the hospital.

*“… when I went home and tried baby on the breast the baby refused at all so like you would put the baby there and they lick the boobs and not suckle at all, so I didn’t breast feed because the baby was not cooperating so I went to therapies [at x hospital] they tried doing the oral massage but would not work.” ****SG_03***

#### Disrupted livelihoods

Extended stays in the neonatal unit with their preterm baby had significant socio-economic impacts for mothers. Having spent weeks or months in hospital with their vulnerable baby, they were reluctant to resume work and leave them at home. For these mothers, going back to work meant separation and breaking the bond they had started creating with the baby. They also feared that other caregivers might not be able to take care of the child as they would.

“*So now the time came when I was just about to go back to work, that bond, it’s not easy to break it. Now you now start having those worries, I have been taking care of my baby all this time and I he had very good weight gain and all that, now I was just wondering, whoever is going to take care of my baby, will this person give him that good attention like maybe I was giving him, will he be able to support him.” ****SG_01***

Some of the women described how their families struggled financially, making it difficult for them to afford the recommended food stuffs for their babies or proper nutrition for the mother, which, in turn, impacted on her ability to breastfeed.

*“… you are supposed to give them fruits, and you don’t have the money for fruits. But when you come to the hospital and they ask you, because you don’t want the doctor to scold you, because you will not start telling them your life story, you are forced to lie, and tell them they eat. If it’s fruits ‘they eat’ and if it is drugs ‘they are taking’. You see, it forces you to just tell them that. To me it has been so hard, but I am struggling. […] So, you see, I skip, I don’t give her the way I am supposed to be giving. But I am struggling to give her. When it comes to feeding the way I am supposed to feed them, I don’t feed them that way, I feed her less. You find that the kilos are not increasing but they are doing well. If it is about doing well, you can see they are okay.” ****HF1_001***

#### Stigma

The previous sections have highlighted the multiple layers of discouragement or lack of confidence that mothers of premature babies experienced. They also described stigma from multiple sources including from health professionals after discharge and their community, as extended family and their community commented on their baby’s low birth weight and delayed developmental milestones. The mothers’ narratives detailed stigma which led to further social isolation. This was compounded by the loss of the community of support that had previously been developed with other mothers facing similar experiences while in the neonatal unit.

*“Taking them outside and sometimes you know it is not like home, here in Nairobi people are many in the residential places. So, you are scared that someone will see them and say they are small”. ****HF1_008***

*“With the family, no [criticism or blame]. with friends, neighbors, general public yes. The first thing everyone sees is gosh, your baby is so tiny, is she not eating? That statement really annoys any preemie mum (laughs) you know. [People give so much advice]: ‘Why are you giving your child so many drugs? when you give them so many drugs, they become immune, they will end up being resistant, you don’t need to give those many drugs’. ‘Oh calcium, go sunbathe her’. So, they become the doctors.” ****SG_05***

Apart from the sensitivity about being told that their babies are tiny, the mothers were also sensitive to comments about their babies’ delayed milestones.

*“’Your baby has not started walking?’ I tell her, “no”. Like I have a neighbour they are one month apart with my son. But you know my son can't walk on his own, but that kid now can walk. He plays outside, mine can’t because he's still scared. Yeah. He can, he will only walk if he's holding something. Yeah. So, someone goes like ah, ‘when was your kid born?’ ‘You know Um, has he not started walking?’ I tell them, no. ‘Why? Mine had already started walking’. Those are things… [laughs] You look at someone and you’re like, you do not know what I’ve been through.” ****SG_12***

These comments from their community contributed to some of the mothers isolating themselves from neighbours, families, and friends.

*“[I didn’t go out] …because you used to meet with someone and they ask you, ‘did you give birth to that small pregnancy?’ And then you know that many questions if you don't want to. I used to lock up myself in the house with my children and because they were very small, I never wanted anyone to see them.” ****HF1_003***

The mothers’ narratives also described the stigmatising behaviour experienced at health facilities. After discharge from the neonatal unit, preterm babies are scheduled for routine follow-up visits to check their progress at either neonatal units or newborn outpatient clinics and routine immunisation/ post-natal clinic visits at the Maternal and Child Health Clinic (MCH). It was in these settings that mothers described experiencing stigma. MCHs cater for all babies and staff were often not privy to the baby’s prematurity and the reasons they were not meeting the expected developmental milestones. Negative comments discouraged the mothers, with some choosing to seek care from alternative facilities.

*When I came back from there eeeh the first day I went to hospital the nurse was like, “you woman, you are not breastfeeding the child, look at how your baby looks he is too young too small at five months you have a baby who looks like a newborn”. And you know I went to the hospital with a very huge file and I just handled over the file and said “This baby was at eight hundred and twenty seven at birth, he is now at three kg that’s a very big progress I think you should be even congratulating me for getting there” and the nurse was like, “You premature mothers think that you’re so special”. I didn’t get, I actually just took my baby and left I didn’t get the service from there, I decided I should go to a private hospital. ****SG_03***

## Discussion

Findings from this study reveal that discharge home after giving birth prematurely was a complex process for the mothers; feelings of joy and fear were juxtaposed against the stress of adapting to life at home away from the hospital setting and medical expertise. Mothers reported their challenges with identifying deterioration; establishing and maintaining feeding and weight gain; the social and economic impacts of a long hospitalisation and looking after a vulnerable premature baby. As has been observed elsewhere [36], stigma experienced in the community and from health professionals involved in follow-up care often exacerbated mothers’ isolation and their anxiety and stress.

Mothers whose babies are admitted in the newborn units do participate in caring for their babies, although the levels of involvement vary. Activities that mothers participate in include wiping/bathing the baby, watching over the baby, breastfeeding and nasogastric tube feeding [8, 37]. However, the amount of support offered to these mothers remains variable and is often insufficient in LMIC settings where staffing levels are a big challenge [38]. As our findings indicate, despite developing skills in the neonatal unit, at the point of discharge mothers had uncertainties about caring for the baby on their own, away from the health professionals and communities of support they had established with other mothers during the admission. These observations about uncertainties have been noted in a UK study on the experiences of parents whose babies have had neonatal surgery and been cared for in a neonatal unit, although these uncertainties receded as parents gained confidence and expertise in caring for their baby at home [39].

We have reported on mothers’ information needs, as well as the range of information they receive on discharge. Information gaps contributed to fears post-discharge. Most of the mothers felt that the information provided pre- and during discharge was enough to support a smooth transition to home. However, as our findings illustrate, mothers experienced mixed emotions at the point of discharge and were often eager to leave the hospital. Even if more time was allocated to discharge information this eagerness may limit their ability to effectively absorb the information. [40, 41]. Additionally, understaffing in the NBUs also hinders staff ability to offer support and adequate information to mothers. Therefore, exploring opportunities for mobile health interventions, where tailored messaging including messages such as danger signs and baby care may be passed on to mothers via phone text messages, could be an alternative option. These approaches are increasingly being taken up to deliver maternal and child health education and promotion in low-income settings [42, 43].

Evidence from both HICs and low-income settings shows that there is a lack of clear discharge guidelines which could help ascertain a mother’s readiness and her capacity to carry on with the baby’s care post-discharge, in particular feeding [44, 45]. Much emphasis is placed on a baby’s clinical indicators such as weight gain [46]. However, our findings highlight difficulties with feeding post-discharge, which might indicate a gap in some mothers’ capacities with this task at the time of discharge. Difficulties in feeding is one of the common challenges in pre-term babies [47], as premature babies often have delayed breastfeeding as they have not developed the ability to suck. The emphasis should therefore be on whether mothers can adequately feed their babies, whether they know how to express and, when their baby develops the ability to suck, whether they know how to initiate breastfeeding. As pointed out by Pérez-Escamilla and colleagues, drawing together evidence from HIC and SSA, health systems have inadequately addressed issues such as mothers’ mental health, anxiety, self-reported insufficient milk and low self-efficacy, all of which affect breastfeeding uptake [48].

Lack of experience and skills to care for premature babies at home are linked to an increased number of hospital readmissions [49, 50]. Oluoch et al. reported that involvement of mothers in the care of the babies while they were still in the neonatal unit helped improve their confidence, making the transition home easier and safer [8]. Involving parents in discharge planning builds confidence in their parenting capabilities and timely information supports the uptake of the parental role as discharge approaches [51]. Our findings confirm that discharge is often handled more as an event than a process, triggered by clinical indicators such as weight gain and weaning from technologies. This may be explained by the realities of neonatal care in Kenya [38]. Research suggests that neonatal units in low-income settings often face service provision bottlenecks such as understaffing and high workloads. Thus developing mothers’ confidence and assessing their competencies is overlooked. Additionally, continuity of care throughout the hospital stay and beyond, including continued provision of information along the continuum after discharge, is important for improved baby mortality and morbidity. Haggerty’s continuity of care framework outlines three categories of continuity, of relevance to post discharge follow-up. These include (i) informational continuity: information being shared and used appropriately; (ii) management continuity: management of illness in a consistent and coherent manner; and (iii) relational continuity: the ongoing relationship between a patient and one or more providers [52].

In these contexts, there is little scope for supporting a multidisciplinary, inter-professional and systematic approach to discharge in which parents have an active role. While not reported extensively in this paper, gender and social norms influence the journeys and care pathways of mothers and their babies. It is thus also important to pay attention to individual and community-level factors such as access to resources, decision-making, autonomy and agency, distribution of labour and social norms which may influence a mother’s ability to practice recommendations made at discharge [28].

Imogen Tyler describes stigma as a practice experienced through looks, comments or remarks that can give rise to shame; “Stigma marks people out: it is intended either by the scar it leaves on the body, or by the spectacle that accompanies it, to brand the victim with infamy’” [53]. In Erving Goffman’s well-known phrase, this becomes a ‘spoiled identity’ [54, 55]. Our findings on the stigma faced by mothers of preterm babies align with findings from other studies conducted in Ghana, Malawi and Uganda [32, 56]. For example, a study conducted by Sakyi et al. on mothers of low birth weight babies in Ghana reported mothers experienced stigma due to the newborns' small size with mothers being blamed and self-isolating in response to the social gossip [57]. The stigma the mothers experienced in these low-income settings – from family members as well as the community at large, where people made comments about how “tiny” the babies looked – has not been widely reported in the literature in high-income settings.

Social stigma can reinforce isolation and structural inequalities and have devastating effects on people’s health and well-being [54]. The different types of social support, such as the peer support highlighted by Oluoch and colleagues, demonstrate that mothers can build mutually supportive relationships in the neonatal unit that can act as a buffer [8, 58]. Yet, in our study, this social capital was lost to some mothers post-discharge. The stigma mothers faced from the community prompted some to hide their babies to avoid comments and questions regarding the baby’s size and development. At the health care facilities during post-natal visits, these mothers faced stigma as they received negative comments from nurses regarding the baby’s weight or developmental milestones, potentially further exacerbating withdrawal from postnatal care. This echoes findings from a study in rural southern Malawi [56] that showed that mothers with low birthweight (LBW) babies felt ashamed and stayed in their homes and did not attend the under-five postnatal clinics as they feared being laughed at or mocked. As noted by Sakyi and colleagues, the stigma associated with small and preterm babies in sub-Saharan Africa not only impacts the parent’s capacity to provide for the child but also impacts their well-being and health [57]. In some settings, premature birth may also be viewed as having an abortion and hence regarded as a “curse” [59].

### Strengths and limitations

We applied rigorous approaches to the collection of mothers’ stories. We used narrative interviewing approaches which allow participants to tell their stories in their own words thus limiting interviewer bias. Additionally, in sampling participants, we aimed for a maximum variation sample, carefully selecting participants across the socio-economic divide in Nairobi, Kenya. We included mothers of preterm babies between 3 months and 2 years old so there was some potential for recall bias although the interviewees rarely lost their train of thought and described their journeys in great detail. Due to the COVID-19 pandemic, three adjustments were made. It was not possible to sample hospitals outside of Nairobi, as had been the original study intention. Some of the interviews were done virtually where the mother was exposed to distractions from the family members, as well as poor internet connection. Women whose babies had died after discharge were not included as researchers felt the study could not provide appropriate trauma support due to the Covid-19 pandemic. The findings of this study cannot be generalised due to the small sample size and being focused on one county in Kenya, however some key lessons and recommendations could be transferable to other similar contexts.

### Recommendations

By examining mothers’ post-discharge experiences, we have highlighted system and individual level challenges which impact not only on mothers’ capabilities and well-being but potentially impact the babies’ well-being and ability to thrive post-discharge. While still in hospital mothers need support to develop skills and confidence. There is a need to recognise the challenges faced by mothers while transitioning home with premature babies and the risks during this period. Strengthening the discharge process through developing guidelines and checklists to support staff is essential, as is thinking through informational and relational continuity and coordination of care and recognising that discharge is a process, not an event. Developing and supporting strategies to understand and manage mothers’ expectations, tackle social stigma, for example, health worker sensitisation, community education and sensitisation on premature babies, should also be prioritised. Training materials based on the research narratives (with video) recorded for this study, could be helpful here for post-natal clinic staff who are seeing, but not specialising, in pre-term infant care. Due to the understaffing and workload considerations, strategies that do not further overburden frontline staff, such as health promotion and text messaging, should be adopted to reach mothers post-discharge with tailored information for ELBW and preterm babies.

## Conclusion

Our findings suggest there is a need to strengthen the timing and adequacy of information and support provided to mothers at the point of discharge at which point co-ordinated post-discharge care service should take over. Targeted engagement interventions could demystify and address knowledge gaps about preterm deliveries at the community level and thereby contribute to tackling the disabling power of social stigma, empowering mothers during these challenging transitions. These findings are important as they could inform strategies for improving mothers’ experiences and maternal competence after neonatal unit discharge and hence the baby’s outcome.

## Supplementary Information

Supplementary Material 1: Table S1. Socio-demographic data of the mothers interviewed.

## Data Availability

The qualitative dataset generated and/or analysed during the current study is not publicly available due the small study sample which makes the participants easily identifiable but are available from the corresponding author on reasonable request.
