# Supplementary material for: After neonatal care, what next? A qualitative study of mothers’ post-discharge experiences after premature birth in Kenya
Source: Int J Equity Health. 2025 Jan 20;24:17. doi: 10.1186/s12939-024-02340-y (PMC11744954; doi:10.1186/s12939-024-02340-y)
Supplement: Supplementary file 1 — Supplementary Material 1: Table S1. Socio-demographic data of the mothers interviewed. [file 12939_2024_2340_MOESM1_ESM.docx]

**Socio-demographic data of the mothers interviewed**

| **Participant ID** | **Age** | **Level of education** | **No of children** | **Admission hospital** |
| --- | --- | --- | --- | --- |
| HF1_001 | 36 | Form 1 | 4 | Public |
| HF1_002 | 19 | Class 7 | 1 | Public |
| HF1_003_ | 29 | Form 4 | 3 | Public |
| HF1_004 | 38 | College | 3 | Public |
| HF1_005 | 23 | College | 2 | Public |
| HF1_006 | 25 | Class 8 | 2 | Public |
| HF1_007 | 25 | Class 8 | 2 | Public |
| HF1_008 | 29 | Form 2 | 2 | Public |
| HF1_009 | 34 | Form 4 | 4 | Public |
| HF1_010 | 18 | Form 3 | 1 | Public |
| HF1_011 | 28 | Form 2 | 2 | Public |
| HF2_001 | 31 | College | 2 | Public |
| HF2_002 | 28 | College | 4 | Public |
| HF2_003 | 39 | Form 3 | 4 | Public |
| HF2_004 | 28 | Form 4 | 2 | Public |
| HF2_005 | 28 | College | 1 | Public |
| HF2_006 | 30 | College | 2 | Public |
| HF2_007 | 34 | Class 7 | 4 | Public |
| HF2_008 | 25 | College | 2 | Public |
| HF2_009 | 37 | Form 4 | 3 | Public |
| HF2_010 | 38 | Class 8 | 3 | Public |
| HF2_011 | 35 | ollege | 4 | Public |
| SG_001 | 32 | Diploma | 3 | Public |
| SG_002 | 28 | Degree | 1 | Private |
| SG_003 | 27 | Diploma | 1 | Private |
| SG_004 | 40 | Degree | 1 | Public |
| SG_005 | 38 | Degree | 1 | Public |
| SG_006 | 34 | Degree | 1 | Private |
| SG_007 | 35 | Masters | 1 | Private |
| SG_008 | 37 | Degree | 3 | Private |
| SG_009 | 37 | Degree | 2 | Private moved to public |
| SG_010 | 34 | Masters | 2 | Private |
| SG_011 | 38 | Degree | 5 | Private moved to public |
| SG_012 | 36 | Degree | 2 | Private |
